# Solid Lipid Nanoparticles Loaded with Dexamethasone Palmitate for Pulmonary Inflammation Treatment by Nebulization Approach

**DOI:** 10.3390/pharmaceutics16070878

**Published:** 2024-06-29

**Authors:** Hsin-Hung Chen, Chen-Hsiang Sang, Chang-Wei Chou, Yi-Ting Lin, Yi-Shou Chang, Hsin-Cheng Chiu

**Affiliations:** 1 Department of Biomedical Engineering and Environmental Sciences, National Tsing Hua University, Hsinchu 30013, Taiwan; bc8472@hotmail.com (H.-H.C.); yishou@gmail.com (Y.-S.C.); 2 Department of R&D, Medical Division, MicroBase Technology Corp, Taoyuan City 33464, Taiwan; samnelsang@microbasetech.com (C.-H.S.); edwinlin@microbasetech.com (Y.-T.L.)

**Keywords:** anti-inflammation, dexamethasone palmitate, nebulization, pneumonia, solid lipid nanoparticles, alveolar macrophages

## Abstract

Pneumonia stands as the leading infectious cause of childhood mortality annually, underscoring its significant impact on pediatric health. Although dexamethasone (DXMS) is effective for treating pulmonary inflammation, its therapeutic potential is compromised by systemic side effects and suboptimal carrier systems. To address this issue, the current study introduces solid lipid nanoparticles encapsulating hydrophobic dexamethasone palmitate (DXMS-Pal-SLNs) as an anti-inflammatory nanoplatform to treat pneumonia. The specialized nanoparticle formulation is characterized by high drug loading efficiency, low drug leakage and excellent colloidal stability in particular during nebulization and is proficiently designed to target alveolar macrophages in deep lung regions via local delivery with the nebulization administration. In vitro analyses revealed substantial reductions in the secretions of tumor necrosis factor-α and interleukin-6 from alveolar macrophages, highlighting the potential efficacy of DXMS-Pal-SLNs in alleviating pneumonia-related inflammation. Similarly, in vivo experiments showed a significant reduction in the levels of these cytokines in the lungs of mice experiencing lipopolysaccharide-induced pulmonary inflammation after the administration of DXMS-Pal-SLNs via nebulization. Furthermore, the study demonstrated that DXMS-Pal-SLNs effectively control acute infections without causing pulmonary infiltration or excessive recruitment of immunocytes in lung tissues. These findings highlight the potential of nebulized DXMS-Pal-SLNs as a promising therapeutic strategy for mitigating pneumonia-related inflammations.

## 1. Introduction

Currently, Currently, pneumonia is the primary infectious cause of child mortality and the sixth leading cause of death across all age groups globally, claiming 2.5 million lives in 2019 [1,2]. In 2020, the worldwide spread of pneumonia attributed to severe acute respiratory syndrome coronavirus 2 (SARS-CoV-2/COVID-19) led to more than 8 million documented cases [3,4]. To address this, the World Health Organization (WHO) and UNICEF have integrated a Global Action Plan for the Prevention and Control of Pneumonia (GAPPD) [5]. Pneumonia often arises from infection caused by various foreign pathogens, including viruses, bacteria and fungi, leading to substantial inflammatory responses in the lung tissues. Individuals with compromised immune function of-ten experience fever and dyspnea as a result of such inflammatory processes [6,7,8]. Severe infections can trigger a dysregulated immune response characterized by excessive secretion of pro-inflammatory cytokines, including TNF-α, IL-6 and IL-1β. Such cytokines induce the infiltration of a large number of immune cells into the patient’s lungs [9,10]. In acute respiratory syndrome cases, such immune cells attack virus-infected lung tissues, resulting in fluid accumulation, swelling, respiratory distress, and even sudden death. Therefore, an effective therapeutic strategy is required to reduce inflammation in the respiratory tract of patients for future SARS-like epidemics.

Previous studies suggest that nebulization is more effective than oral or intravenous routes for delivering anti-inflammatory drugs to treat inflammation induced by bacterial or viral infections to the lungs, such as bronchitis, pneumonia, etc. [11,12,13]. This administration route offers advantages, such as quick delivery to inflamed lung regions, a rapid onset of therapeutic effect, and a reduction in drug dosage requirement [14]. However, the limited aqueous solubility of some drugs can lead to crystallization or precipitation during nebulization, hindering efficient delivery to the lungs [15]. To mitigate such drawbacks, encapsulating drugs within solid lipid nanoparticles (SLNs) has emerged as a method of creating promising nanocarriers for efficient local drug delivery to lung regions [16]. SLNs are submicron-sized particles used as a colloidal drug delivery system, which serve as an alternative to traditional carriers such as polymeric micelles, liposomes, etc. [17,18]. The lipid matrix of SLNs safeguards the encapsulated drugs against degradation, oxidation, or hydrolysis, thus improving stability during storage and administration. Additionally, SLN-encapsulated drugs via nebulization delivery for the treatment of pneumonia and/or other pulmonary diseases offer several advantages, including improved local delivery to lungs, enhancement of drug bioavailability, potential anti-inflammatory action, and a reduction in pulmonary emphysema [19,20,21]. However, the pulmonary drug delivery systems based upon the SLN formulation via nebulization administration in the treatment of pulmonary inflammation were barely reported [22]. In particular, the evaluation of the in vivo therapeutic efficacy of SLN-based anti-inflammatory therapeutics for local lung delivery via the nebulization process in the absence of animal anaesthetization was not found.

Dexamethasone (DXMS) Dexamethasone (DXMS) is a widely employed corticosteroid for the treatment of various acute inflammations and tissue injuries, including rheumatoid arthritis, pneumonia, dermatitis, allergies, asthma, surgical site infection, chronic obstructive pulmonary disease [23,24] and cytokine storm [25]. Although the side effects of DXMS may not be immediately evident, common adverse reactions such as dyspepsia, insomnia, bitter taste, nausea, and headache have been reported [26]. DXMS functions as a glucocorticoid receptor agonist and has demonstrated high efficacy in hospitalized COVID-19 patients in need of respiratory support [27,28]. Upon binding to glucocorticoid receptors on immunocytes, DXMS downregulates the gene expression of pro-inflammatory cytokines, thereby reducing inflammation at the infection site [29]. Dexamethasone sodium phosphate (DSP), a water-soluble prodrug of DXMS, is often used in injectable forms, such as intravenous, intramuscular, and oral administration, due to the poor water solubility of DXMS [30,31]. However, integrating hydrophilic DSP into the hydrophobic core of nanomedicine delivery systems poses challenges, leading to diminished drug loading capacity and increased drug leakage. Dexamethasone palmitate (DXMS-Pal), a hydrophobic compound and a prodrug of DXMS, undergoes conversion to DXMS via esterase metabolism [32,33]. DXMS, frequently used for its immunosuppressive properties in the treatment of inflammatory diseases, has attracted significant attention in recent nanotechnology research owing to the facile uptake of the nanoparticle-based drug formulation by macrophages or other immunocytes via phagocytosis [34,35]. Macrophages, known for their inflammatory responses in secreting proinflammatory cytokines to bacterial/viral infections, are, therefore, a good target for the treatment of inflammation [36,37]. To the best of our knowledge, no studies have yet reported on SLNs loaded with DXMS-Pal and their efficacy in treating pulmonary inflammation through nebulization, both in vivo and in vitro.

**Scheme 1:**
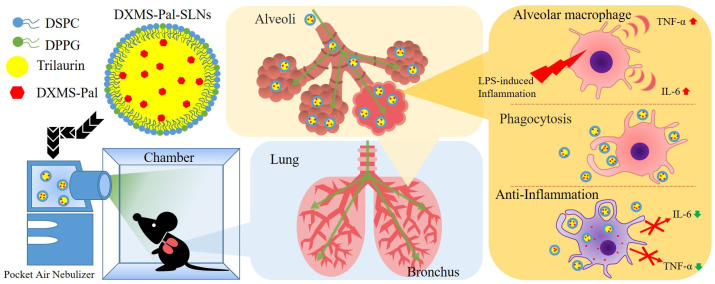
Schematic representation of the working mechanism of nebulized DXMS-Pal-SLNs for the treatment of pneumonia-related inflammation.

In the present study, SLNs containing DXMS-palmitate (DXMS-Pal-SLNs) were developed by the emulsion evaporation technique and evaluated on their efficacy via nebulization delivery for pulmonary inflammation treatment. DXMS-Pal is a covalent conjugate of dexamethasone with palmitic acid via an ester linkage, rendering the prodrug more nonpolar than the parent drug and thus facilitating its encapsulation into SLNs. The in vitro anti-inflammatory effects of the formulated DXMS-Pal-SLNs on MH-S cells in terms of cellular uptake and cytokine secretion (TNF-α and IL-6) were evaluated (Scheme 1). The LPS-induced pulmonary inflammation in C57BL/6JNarl mice was established and employed for the in vivo characterization of the DXMS-Pal-SLNs in suppressing the inflammatory responses. The in vivo therapeutic efficacy, in terms of targeted delivery to lung regions and inhibition of proinflammatory cytokine secretion, was investigated with the local delivery of DXMS-Pal-SLNs via nebulization attained from a mesh type nebulizer producing mist with median mass aerodynamic diameter (MMAD) < 5 μm as a facile and effective approach to the transportation of the SLN formulation.

## 2. Materials and Methods

### 2.1. Materials

Trilaurin, 1,2-distearoyl-sn-glycero-3-phosphocholine (DSPC), 1,2-dipalmitoyl-sn-glycero-3-phosphoglycerol (DPPG), dexamethasone palmitate (DXMS-Pal), lipopolysaccharide (LPS), and Hoechst 33258 were purchased from Sigma-Aldrich, St. Louis, USA. Dexamethasone sodium phosphate (DSP) was obtained from Tianjin Tianyao Pharmaceutical Co. Ltd. Tianjin, China. All solvents were supplied by TEDIA, Fairfield, USA. Pocket Air Nebulizer (MBPN002) was provided by Microbase Technology Corp., Taoyuan City, Taiwan, through cooperation under Contract Number 109A0153K6. The 3-(4,5-Dimethylthiazol-2-yl)-2,5-diphenyltetrazolium bromide (MTT), Dulbecco’s modified Eagle’s medium (DMEM), ELISA kits (for TNF-α and IL-6), 0.25% trypsin-EDTA, penicillin-streptomycin solution, fetal bovine serum (FBS), and 1,1′-dioctadecyl-3,3,3′,3′-tetramethylindocarbocyanine perchlorate (DiI) were purchased from Thermo Fisher Scientific, Waltham, MA, USA. Murine alveolar MH-S macrophages were provided by the Food Industry Research and Development Institute (Hsinchu City, Taiwan). Male C57BL/6JNarl mice were purchased from the National Laboratory Animal Center, Taiwan.

### 2.2. Preparation and Characterization of DXMS-Pal-SLNs

DXMS-Pal-SLNs were prepared by an emulsion-evaporation process [38]. Briefly, trilaurin (20 mg), DSPC (4 mg), DPPG (1 mg) and DXMS-Pal (4, 6, and 8 mg, respectively) were added to chloroform (1.0 mL). The solution was subjected to vigorous agitation via an ultrasonication probe (Vibra Cell, Sonics, model: VCx750, power: 750 W, powersonic410, Hwashin, Republic of Korea) for 10 min. Subsequently, phosphate buffer (2.5 mL, pH 7.4, ionic strength 10 mM) was added, followed by another ultrasonic homogenization for 10 min. After that, the chloroform was eliminated from the emulsion using rotary evaporation. The product underwent purification via dialysis (MWCO 12–14 kDa) in phosphate buffer (pH 7.4) for 4 days and was collected by lyophilization. The purity was checked and confirmed by Sephadex G-25 PD-10 column (1 cm × 5 cm; gravity flow) with elution against DI water (10 mL) and ethanol (20 mL) in sequence.

The encapsulation efficiency of DXMS-Pal in DXMS-Pal-SLNs was determined by the dissolution of DXMS-Pal-SLNs in ethanol, and the absorption at 238 nm was evaluated using a UV/Vis spectrometer (U-2900, Hitachi, Japan). DXMS-Pal was used as the standard sample to establish the calibration curve in ethanol. Mean particle size, zeta potential and polydispersity index were examined by dynamic light scattering (DLS, Zetasizer Nano ZS, Malvern, UK) measurements employing a Malvern ZetaSizer Nano Series. The morphology of the DXMS-Pal-SLNs was studied with transmission electron microscopy (TEM) (JEOL JEM-2100F, JEOL, Japan).

### 2.3. Drug Leakage Evaluation of SLNs

To evaluate the drug leakage of SLNs in a physiological environment, the aqueous solution of drug-loaded SLNs in phosphate buffer saline (PBS) (pH 7.4; 1.0 mL; 0.5 mg/mL) was placed in a dialysis bag (MWCO 12–14 kDa) against PBS (40 mL) at 37 °C under shaking (reciprocal shaking 100 rpm) for different time intervals. At prescribed time intervals, the nanoparticle (NP) solution in dialysis tubing was collected and lyophilized. The sample was dissolved in ethanol (2.0 mL). The resulting solution was then analyzed by UV/Vis absorption spectroscopy at 238 nm to quantify the levels of DXMS-Pal remaining in NPs and the leakage portion. The drug leakage from DXMS-Pal-SLNs was also examined by the gel permeation chromatography (Sephadex G-25 PD-10 column) with elution against PBS and ethanol in sequence after the incubation of DXMS-Pal-SLNs in PBS at 37 °C for 24 h. The leakage and retention levels were determined by UV/Vis spectroscopy at 238 nm.

### 2.4. In Vitro Analysis

#### 2.4.1. Uptake Study

MH-S cells were seeded onto a 24-well plate at a density of 2 × 10^4^ cells per well, followed by incubation for 24 h at 37 °C in a humidified atmosphere containing 5% CO_2_. Subsequently, cells were treated with DiI-labeled DXMS-Pal-SLNs (DiI concentration 1.6 μg/mL, DXMS concentration 10 μM) for prescribed time intervals. After the cells were being washed with PBS, DMSO was added for cell lysis. The intracellular fluorescence signal intensity of DiI as a fluorescence probe was quantitatively assessed using a microplate reader (FLUOstar OPTIMA, BGM Labtech, Germany). For the cellular uptake examination by automated cell imaging system (ImageXpress Pico, Molecular Device, USA), MH-S cells were seeded on 22 × 22 mm^2^ glass coverslips in 6-well plates (5.0 × 10^5^ cells/well) and incubated at 37 °C for 24 h. Cells were then treated with DiI-DXMS-Pal-SLNs for 4 h and washed thrice with PBS. Hoechst 33342 was employed to stain the cell nuclei. The intracellular distribution of DiI was examined through fluorescence microscopy with excitation and emission wavelengths at 550 and 565 nm, respectively.

#### 2.4.2. Cytotoxicity

MH-S cells were seeded into 96-well plates (6 × 10^3^ cells/well) in DMEM supplemented with 10% FBS and 1% penicillin and incubated for 24 h at 37 °C. Following the removal of the spent medium, fresh DMEM (100 μL) containing either DSP or DXMS-Pal-SLNs was added to each well and co-incubated for 24 h. After being rinsed several times with PBS, cells were re-incubated for another 24 h. MTT solution (10 μL; 5.0 mg/mL) was added and incubated for 4 h at 37 °C. Absorbance was measured at 570 nm by the microplate reader after replacing the culture medium with DMSO (100 µL).

#### 2.4.3. In Vitro Anti-Inflammatory Efficacy

To investigate the anti-inflammatory properties of DXMS-Pal-SLNs in vitro, ELISA kits were used to quantify the levels of two inflammatory cytokines, namely TNF-α and IL-6, in MH-S cells. A density of 8.0 × 10^4^ cells per well was used to seed cells in 24-well plates, followed by incubation for 24 h at 37 °C. The spent medium was then replaced with fresh medium containing LPS (0.1 μg/mL) and incubated for 4 h at 37 °C. After being rinsed with PBS several times, cells were treated with DSP, DXMS-Pal-SLNs-1 and DXMS-Pal-SLNs-2 of three different DXMS concentrations (0.1, 1.0 and 10.0 µM), respectively, for 3 h. Following incubation, the culture medium was collected, and the concentrations of TNF-α and IL-6 were quantified using commercially available ELISA kits (Thermo Fisher Scientific, Waltham, MA, USA) according to the manufacturer’s protocols.

### 2.5. In Vivo Study

#### 2.5.1. Animal Model

All animal experiments were conducted on male C57BL/6JNarl mice, aged 7 weeks, from the National Laboratory Animal Center of Taiwan. All animal experiments adhered to the guidelines set forth by the National Tsing Hua University Institutional Animal Care and Use Committee (IACUC approval number: 109053). Animals were anesthetized using a Zoletil-Rompun combination to ensure minimal pain perception during surgical procedures. Surgical tracheotomies were conducted under anesthesia, followed by the induction of pulmonary inflammation through intratracheal administration of LPS derived from Escherichia coli, as depicted in Figure S1. The LPS dose of either 1 mg/kg or 2 mg/kg in PBS (100 μL) was employed. Following surgery and LPS administration, wound sites were sutured and disinfected with iodine tincture and 75% ethanol.

#### 2.5.2. Administration of DXMS-Pal-SLNs Via Intratracheal Injection

Therapeutic formulations of DSP and DXMS-Pal-SLNs in PBS (100 μL) were directly administered into the mice trachea at a dose of 5 mg/kg of DXMS at 2 h post LPS treatment. After 22 h of administration, the mice were euthanized and bronchoalveolar lavage fluid (BALF) containing alveolar macrophages and other immunocytes was collected by the perfusion of 1.0 mL PBS using an intravascular catheter. The levels of pro-inflammatory cytokines, specifically TNF-α and IL-6, in the BALF were quantified using ELISA kits [39].

#### 2.5.3. Administration of DXMS-Pal-SLNs Via Nebulization

At 6 h after the LPS administration, the mice were placed in a closed chamber with a volume of 13 cm^3^. Formulations composed of DSP and DXMS-Pal-SLNs with varying DXMS doses (5, 10, and 15 mg/kg) in PBS (6.0 mL), respectively, were aerosolized into the sealed chamber using a Pocket Air nebulizer (MBPN002, Taoyuan City, Taiwan). The nebulizer has been commonly employed in clinical settings for treating various respiratory conditions [40,41]. The nebulization process lasted for about 20 min. The mice were euthanized in a CO_2_ chamber at 42 h after the nebulization, followed by lung perfusion with PBS (1.0 mL) through an intravascular catheter. The BALF was collected and the levels of pro-inflammatory cytokines, namely TNF-α and IL-6, were assessed with ELISA kits.

#### 2.5.4. H and E Staining

Tissue hematoxylin and eosin (H and E) staining was conducted according to the protocol delineated by Ruan et al. (2013) [42]. Following the treatment, the major organs were harvested from the euthanized mice, embedded in OCT, and stored at −85 °C for subsequent H and E staining. Histological evaluations of the stained tissues were performed using a 20× microscope (Olympus IX70, Nottingham, NH, USA).

#### 2.5.5. Hemolysis Test

The hemolysis assay was examined according to the procedure established by Khullar et al. [43]. Whole blood was collected from 7-week-old mice through submandibular bleeding. Each 100 μL aliquot of the collected blood was diluted 1:9 with PBS, deionized water, or SLNs in PBS (0.35 mg/mL). After being incubated at 37 °C for 6 h, samples were centrifuged at 7000 rpm, and the integrity of the red blood cell pellet was subsequently assessed.

### 2.6. Statistical Analysis

All experiments in this study were conducted at least in triplicates and data were presented as mean ± standard deviation (SD). The level of statistical significance was determined using a student’s *t*-test. Statistical significance is indicated as (not significant, n.s.) *p* > 0.05, (*) *p* < 0.05, and (**) *p* < 0.01.

## 3. Results and Discussion

### 3.1. Characterization of DXMS-Pal-SLNs

**Figure 1:**
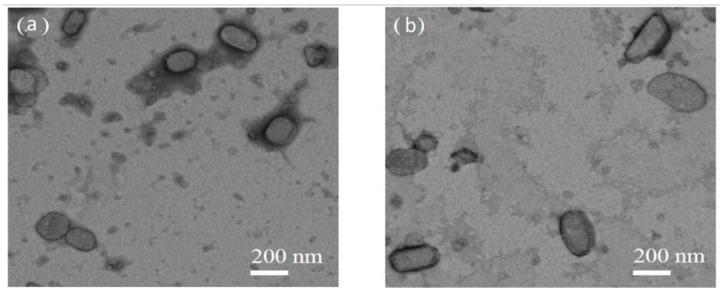
TEM images of (**a**) DXMS-Pal-SLNs-1 and (**b**) DXMS-Pal-SLNs-2.

DXMS-Pal-SLNs were prepared by the emulsion-evaporation technique [38]. Trilaurin was used as the major matrix component of the SLNs, with the incorporation of DSPC and DPPG acting as the colloidal stabilizer, while DXMS-Pal was encapsulated within the solid cores by the hydrophobic association. Three different DXMS-Pal SLNs were prepared with varying contents of DXMS-Pal (namely, 15.8, 23.3 and 26.3 wt%). The mean hydrodynamic size, polydispersity, zeta potential, loading efficiency and content of DXMS-Pal of the DXMS-Pal-SLNs are summarized in Table 1. The mean hydrodynamic diameter (Dh) and polydispersity index (PDI) of three DXMS-Pal-SLNs in phosphate buffer at pH 7.4 were all rather close, being in the range 183–196 nm in diameter along with PDI at 0.2, signifying that the mean hydrodynamic size and the size distribution were not significantly affected by drug encapsulation. DXMS-Pal-SLNs-1 and DXMS-Pal-SLNs-2 in phosphate buffer exhibited good colloidal stability over 3 days at 37 °C and 14 days at 4 °C, with changes in D_h_ less than 10%. This is ascribed to the high negative zeta potential values between −45.5 mV and −47.9 mV by the incorporation of DPPG on the NP surfaces. In addition, the Zwitterionic effect of the DSPC species on anti-aggregation/fouling may also account in part for its prominent colloidal stability in the aqueous phase. The consistency in high negative zeta potential also signifies the presence of monodispersed formulations devoid of aggregation [44]. In contrast, the significantly lower zeta potential value of DXMS-Pal-SLNs-3 compared to DXMS-Pal-SLNs-1 and DXMS-Pal-SLNs-2 implies reduced inter-particulate electro-static repulsion, rendering the NPs likely to aggregate as being observed in phosphate buffer at 4 °C over 7 days (Table 1). Notably, a high encapsulation efficiency of DXMS-Pal into the SLNs (>93.9%) was attained. In comparison with the loading efficiency of DXMS at only approximately 60% into the SLNs, the appreciably increased encapsulation efficiency of DXMS-Pal is obviously caused by the conjugation of the parent drug with palmitic acid that enhances the hydrophobic nature of the payload and facilitates encapsulation into SLNs by hydrophobic effect. In addition to a reduced loading efficiency, the DXMS-SLN formulation was not adopted in this work mainly because of a fast leakage behavior of DXMS (80% within 4 h) from the SLNs in PBS (data shown below). Table 1 shows both the loading content of DXMS-Pal serving as a hydrophobic DXMS prodrug and the equivalent content of the DXMS parent drug in all three DXMS-Pal-SLNs for easy comparison. Unfortunately, precipitates/aggregates were observed from the aqueous DXMS-Pal-SLN-3 dispersion in the phosphate buffer at 4 °C over 7 days, probably due to the overloading of the prodrug deteriorating charge repulsive interactions among NPs. As a result, only DXMS-Pal-SLNs-1 and DXMS-Pal-SLNs-2 were employed for further characterization and evaluation of the therapeutic performance of their applications against pneumonia-related inflammation via nebulization administration. The size and morphology of the drug-loaded SLNs were also examined by TEM. The TEM images in Figure 1a,b show the average size of DXMS-Pal-SLNs below 200 nm. The NPs were not significantly changed in size with the different amounts of DXMS-Pal being incorporated. However, due to the soft structural characteristic of SLNs assembled mainly from small molecular lipids, the NPs were apt to become rather flattened and irregular in morphology during the TEM sample preparation and thus different from the SLN morphology in aqueous dispersion [17].

The performance of the DXMS-Pal-SLNs in combination with a mesh type nebulizer for the nebulization administration was preliminarily evaluated by the recovery rate and morphologic changes of the SLN after the nebulization process. The SLNs in phosphate buffer were introduced into the nebulizer chamber and the emitted SLNs were collected. As shown in Table 2, the recovery rates of the SLNs after nebulization were as high as about 90% and 86% for DXMS-Pal-SLNs-1 and DXMS-Pal-SLNs-2, respectively. Furthermore, negligible changes in both particle size and PDI of the DXMS-Pal-SLNs were observed. The data strongly indicate that the SLNs were barely damaged by nebulization while an excellent spraying performance of the SLNs with the nebulizer was attained.

### 3.2. Drug Leakage Behaviors of Drug-Loaded SLNs

Drug leakage of DXMS-Pal-SLNs and DXMS-SLNs in PBS at pH 7.4 at 37 °C was evaluated. It is noteworthy that the NP-based therapeutic strategy in this work intends to locally deliver the therapeutic agent in the SLN formulation to alveolar macrophages for the pulmonary inflammation treatment. Taking advantage of the prominent phagocytosis activity of (alveolar) macrophages compared to the endocytosis by other non-phagocytic cells, the increased accumulation of the SLNs in macrophages can thus be expected. By contrast, the parent drug, DXMS, or a prodrug such as DSP, when used alone in the absence of the NP formulation penetrates into cells by passive diffusion without selectivity. The drug release from the DXMS-Pal-SLNs for the onset of therapeutic action relies mainly on the degradation of the SLNs and the activation of DXMS-Pal into DXMS via hydrolysis of the ester linkage occurring intracellularly (primarily in lysosomes) after phagocytosis of the NPs by alveolar macrophages. It is therefore considered as drug leakage, with the drug being released from the SLNs extracellularly. The results illustrated in Figure S2A indicate that approximately 86% of the encapsulated DXMS was released from the DXMS-SLNs within the initial 4 h and reached approximately 90% within 24 h. Conversely, the DXMS-Pal leakage from DXMS-Pal-SLNs was dramatically reduced, reaching a plateau level of approximately 15% over 48 h (Figure S2B). The highly reduced leakage of DXMS-Pal was obviously caused by the strong association with SLNs by hydrophobic effect, thereby providing increased cargo content confined within SLNs toward macrophages and possibly reducing unintended side effects. To further investigate the drug leakage characteristics of DXMS-Pal-SLNs, these NPs were subjected to incubation in PBS at 37 °C for 24 h and size-exclusion elution through a Sephadex G-25 PD-10 column. As outlined in Table S1, post-elution retention of DXMS-Pal in the SLN formulations ranged from 86.3% to 93.5%, corroborating the strong association between DXMS-Pal and SLNs mediated by hydrophobic interactions. Therefore, the SLNs with high drug loading can effectively target alveolar macrophages in inflamed regions of the lungs with minimal leakage, thus increasing the therapeutic efficacy in local pulmonary inflammation treatment.

### 3.3. In Vitro Cellular Uptake, Cytotoxicity and Anti-Inflammatory Efficacy

**Figure 2:**
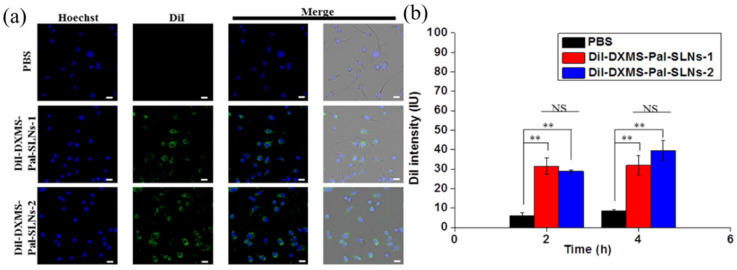
(**a**) Fluorescence images of MH-S cells co-incubated with DiI-DXMS-Pal-SLNs for 4 h at 37 °C. Scale bars are 20 μm. DiI was employed as a fluorescence probe for monitoring the cellular uptake of the SLNs by the alveolar macrophages. The nuclei were stained with Hoechst 33342 in blue. (**b**) Intracellular DiI fluorescence intensity of MH-S cells after co-incubation with DiI-DXMS-Pal-SLNs for 2 h and 4 h, respectively, followed by the cell lysis with DMSO (*n* = 3). The DiI fluorescence intensity was determined by fluorescence spectroscopy. NS *p* > 0.05 and ** *p* < 0.01.

The cellular uptake of DiI-labeled DXMS-Pal-SLNs (DiI-DXMS-Pal-SLNs) was analyzed in MH-S cells. DiI was used to serve as a fluorescent probe for examination of their cellular uptake by the alveolar macrophages. Figure 2a shows the fluorescence images of the MH-S cells after co-incubation with DXMS-Pal-SLNs for 4 h at 37 °C. Significant DiI fluorescence signals were observed for both DXMS-Pal-SLNs-1 and DXMS-Pal-SLNs-2 within the MH-S cells, as compared to PBS. To further examine the cellular uptake of the drug-loaded NPs, the intracellular DiI-DXMS-Pal-SLNs levels after their reaction with MH-S cells for 2 and 4 h were evaluated by determining the DiI fluorescence intensity via fluorescence spectroscopic measurements, following the cell lysis with DMSO. The results shown in Figure 2b demonstrate similar cellular uptake between DiI-DXMS-Pal-SLNs-1 and DiI-DXMS-Pal-SLNs-2 up to 4 h co-incubation. The results clearly signify the substantial uptake of DiI-DXMS-Pal-SLNs by alveolar macrophages through phagocytosis, which leads to enhanced anti-inflammatory efficacy.

**Figure 3:**
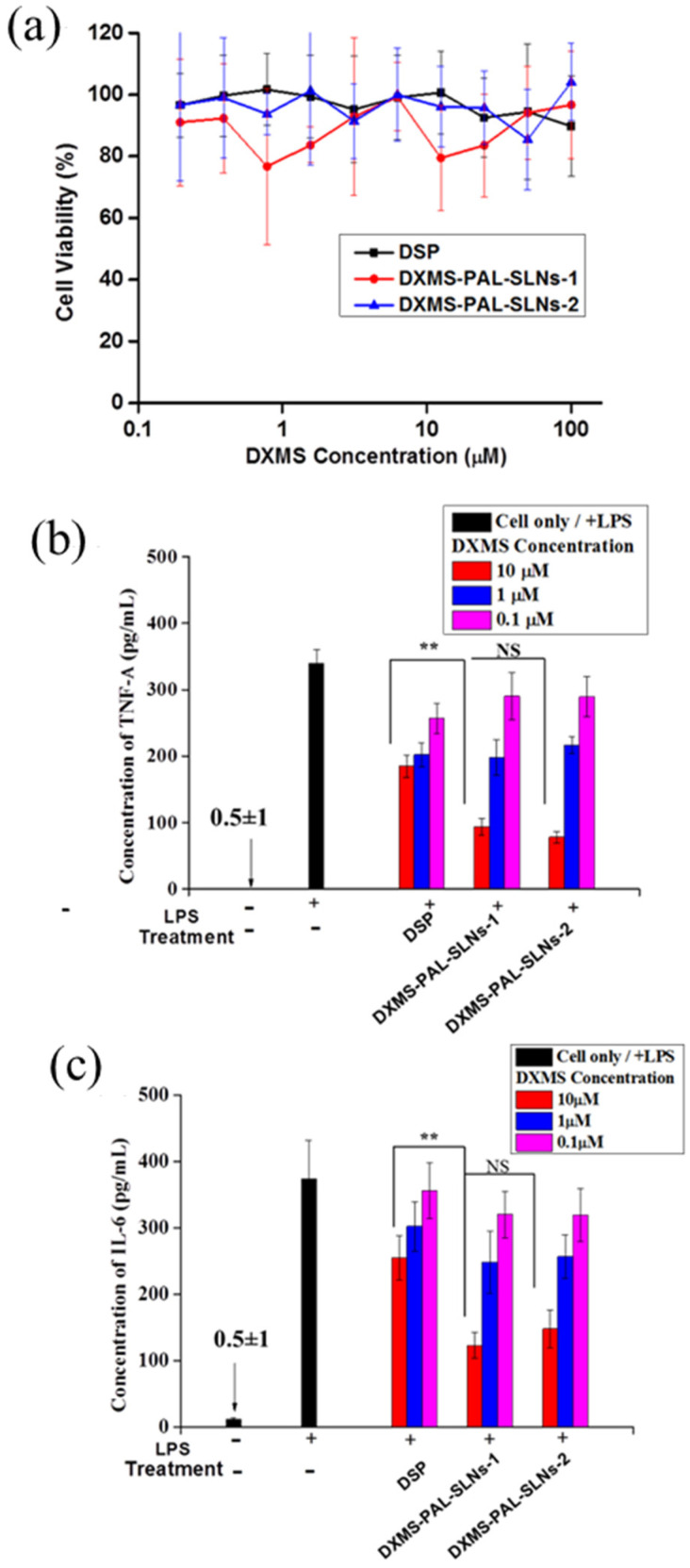
(**a**) Cell viability of MH-S cells treated with DSP, DXMS-Pal-SLNs-1 and DXMS-Pal-SLNs-2 by MTT assay (*n* = 3). The secretion levels of (**b**) TNF-α and (**c**) IL-6 in LPS-induced inflammatory MH-S cells after the treatment with DSP and DXMS-Pal-SLNs at various DXMS concentrations (*n* = 3). The cytokine levels were determined by ELISA kits. NS *p* > 0.05 and ** *p* < 0.01.

The MTT assay was utilized to study the in vitro cytotoxicity of DSP and DXMS-Pal-SLNs. As DXMS has a low aqueous solubility affecting the cellular uptake, DSP as a hydrophilic derivative of DXMS that has been often clinically employed was used as a control sample. The results as shown in Figure 3a indicate no substantial reduction in cell viability of MH-S cells by DXMS-Pal-SLNs and DSP after 24 h treatment, indicating the negligible toxic nature of DSP and DXMS-Pal-SLNs at the dose up to 100 μM. To examine the in vitro anti-inflammatory efficacy of DXMS-Pal-SLNs, MH S cells were first treated with LPS and subsequently co-incubated with DSP, DXMS-Pal-SLNs-1 and DXMS-Pal-SLNs-2 for 24 h, respectively. The inflammatory cytokine levels, specifically TNF-α and IL-6, were quantified using ELISA kits. As shown in Figure 3b,c, the intracellular levels of TNF-α and IL-6 were increased to about 340 pg/mL and 374 pg/mL, respectively, with the LPS stimulation, yet in the void of the anti-inflammation treatment. Treatments with DSP, DXMS-Pal-SLNs-1 and DXMS-Pal-SLNs-2 at a dose of 0.1 µM show only slight effects to reduce the inflammation. With the dose of DSP being enhanced to 10 μM, both the levels of TNF-α and IL-6 were reduced to 185 pg/mL and 255 pg/mL, respectively. While the dose was increased to 10 μM for both DXMS-Pal-SLNs-1 and DXMS-Pal-SLNs-2, the TNF-α levels were appreciably reduced to about 96.29 and 77.27 pg/mL. Similarly, the IL-6 concentrations were found to be 125.21 and 150.32 pg/mL separately by the treatments of DXMS-Pal-SLNs-1 and DXMS-Pal-SLNs-2 at the dose of 10 μM. The decreased therapeutic effect of DSP in the anti-inflammation performance compared to the DXMS-Pal-SLN counterparts is most probably caused by the reduced cellular uptake via passive diffusion due to its hydrophilic nature and its rapid metabolic degradation within the cells [45]. By contrast, the pronounced reduction in the levels of pro-inflammatory cytokines (i.e., TNF-α and IL-6) was observed with the treatment of LPS-pretreated MH-S cells with DXMS-Pal-SLNs. The results are ascribed to the satisfactory amounts of DXMS-Pal-SLNs internalized by the cells via phagocytosis and of the DXMS parent drug by the release of DXPS-Pal from SLNs in lysosomes and activation of DXMS-Pal into DXMS by ester hydrolysis in lysosomes or cytoplasm. It has been reported that DXMS-Pal can undergo intracellular ester hydrolysis, giving rise to DXMS, either chemically or enzymatically [32]. The in vitro characterization shows that DXMS-Pal-SLNs developed in this work are not cytotoxic, yet are capable of being engulfed into alveolar macrophages via phagocytosis and inhibiting the secretion of inflammatory cytokines such as TNF-α and IL-6 efficiently.

### 3.4. In Vivo Anti-Inflammatory Efficacy

**Figure 4:**
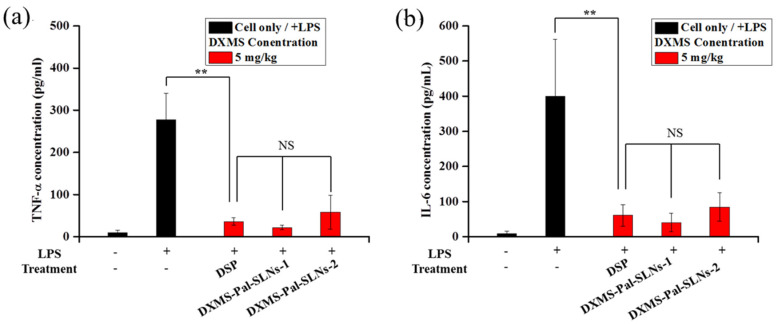
Determination of the levels of (**a**) TNF-α and (**b**) IL-6 in the BALF of mice experiencing LPS-induced lung inflammation (LPS 1.0 mg/kg in 100 μL of PBS) at 22 h after the treatments with DXMS-Pal-SLNs and DSP (DXMS 5.0 mg/kg in 100 μL of PBS), respectively, via intratracheal injection (*n* = 6). The cytokine levels were determined by ELISA kits. NS *p* > 0.05 and ** *p* < 0.01.

The objective of this study was to assess the efficacy of DXMS-Pal-SLNs via nebulization administration in mitigating the release of inflammatory cytokines, specifically TNF-α and IL-6, implicated in the progression of pneumonia. LPS has been frequently used to induce acute pulmonary inflammation in establishing an animal pneumonia model [46,47,48]. Male mice with LPS-induced pulmonary inflammation were employed to serve as the in vivo animal model. Eliciting the efficacy of DXMS-Pal-SLNs via nebulization approach was attained mainly by comparing the data with those via intratracheal injection as the latter directly delivers the therapeutics into the lungs and, therefore, maximizes the anti-inflammatory effect. DSP was employed as a control sample and ELISA kits were used to determine the concentrations of TNF-α and IL-6 quantitatively in the BALF for each group. To prevent the mice from drowning, the administration of the solutions of LPS (1.0 mg/kg) and therapeutic agents in PBS was limited to a total volume of 100 μL. Following intratracheal administration, asphyxiated mice were immediately provided with respiratory support through pure O_2_ for 30 min. The data shown in Figure 4a,b reveal that mice treated with only LPS exhibit the highest levels of TNF-α and IL-6, with the concentrations reaching approximately 277 pg/mL and 400 pg/mL, respectively. Conversely, treatment with DSP and DXMS-Pal-SLNs by intratracheal injection significantly reduced the levels of both cytokines. Following DSP treatment, the released concentrations of TNF-α and IL-6 were measured to be 36 pg/mL and 60 pg/mL, respectively. Similarly, DXMS-Pal-SLNs-1 effectively reduced TNF-α and IL-6 release, with the concentrations being 22 pg/mL and 41 pg/mL, respectively. The DXMS-Pal-SLNs-2 treatment also reduced cytokine secretion levels, with the measured TNF-α and IL-6 concentrations at 58 pg/mL and 85 pg/mL, respectively. Thus, no statistically significant difference in anti-inflammatory effect was observed among the DSP and two DXMS-Pal-SLNs groups after the treatment, confirming the pronounced anti-inflammatory effect of DXMS-Pal-SLNs is comparable with DSP when administered via intratracheal injection.

**Figure 5:**
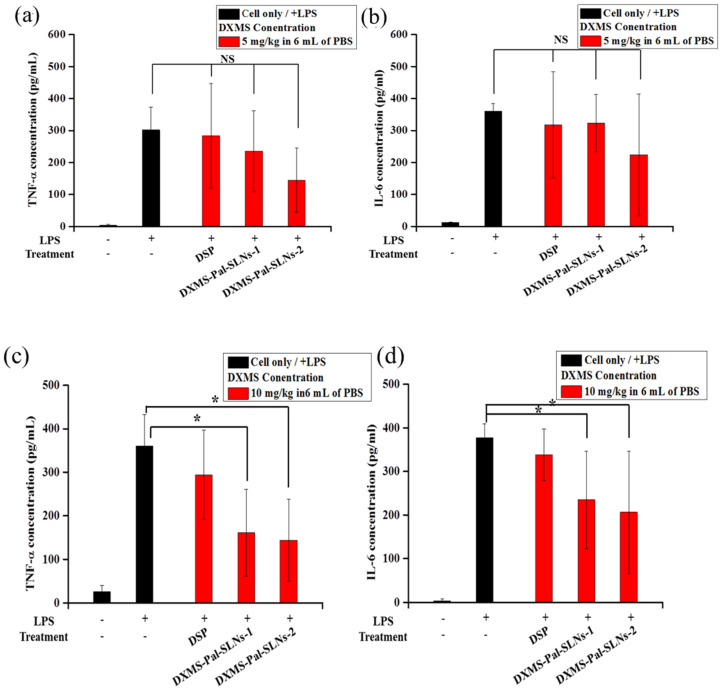

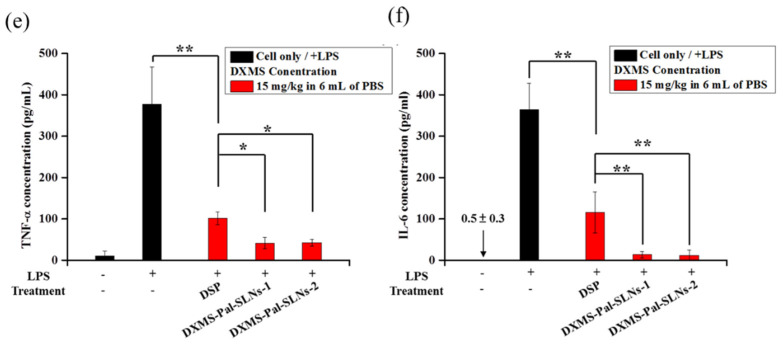
Determination of the levels of TNF-α and IL-6 in the BALF of mice experiencing the LPS-induced lung inflammation (LPS 1.0 mg/kg) at 22 h after the treatments with DXMS-Pal-SLNs and DSP, respectively, via the nebulization administration (*n* = 6) at the DXMS doses of (**a**,**b**) 5.0 mg/kg, (**c**,**d**) 10.0 mg/kg and (**e**,**f**) 15.0 mg/kg in 6 mL of PBS. The cytokine levels were evaluated by ELISA kits. NS *p* > 0.05, * *p* < 0.05 and ** *p* < 0.01.

Mice with LPS-induced pulmonary inflammation were also treated with DSP and DXMS-Pal-SLNs, respectively, at the same DXMS doses by nebulization administration using a mesh type Pocket Air nebulizer producing mist with the MMAD smaller than 5 μm. Three DXMS doses were employed, namely 5.0, 10.0 and 15.0 mg/kg. The DXMS-Pal-SLNs in PBS with the total volume of 6.0 mL was used for nebulization over the duration of 20 min. The levels of TNF-α and IL-6 in the BALF were evaluated by ELISA kits. The data are illustrated in Figure 5. As indicated in Figure 5a,b, DXMS-Pal-SLNs and DSP at a DXMS dose of 5.0 mg/kg were not effective in inhibiting TNF-α and IL-6 secretions from alveolar macrophages. Statistically significant differences in both cytokine levels among DSP, DXMS-Pal-SLNs-1, and DXMS-Pal-SLNs-2 were also not attained. The results clearly indicate that the DXMS dose of 5.0 mg/kg in DSP and SLN formulations via the nebulization administration designed in this work was too low to show any significant changes in both the cytokine levels, although the same dose administered via the intratracheal injection exhibited prominent inhibitory effect on inflammation by alleviating the TNF-α and IL-6 secretions. While it is anticipated that the bioavailability via the nebulization administration be appreciably reduced compared to the intratracheal injection, the therapeutic effect can be expectedly promoted with an appropriate nebulizer mask being adopted instead of a closed chamber where the mice were restrained. However, the mice mask is inaccessible and it is rather difficult for mice to wear the mask without anesthesia because it requires them to keep their breath as steadily as possible. With the dose of DXMS being increased to 10 mg/kg, the reduction in the levels of both TNF-α and IL-6 by the treatments with both DXMS-Pal-SLNs became rather significant, while the decreased effects of the DSP treatment on alleviating the secretion of the pro-inflammatory cytokines at this dose compared to the SLNs was observed (Figure 5c,d). The results indicate the superior therapeutic efficacy by the SLN formulation over the simple hydrophilic prodrug (DSP) via the nebulization administration route. With the dose being further increased to 15 mg/kg, the nebulization administration of DXMS-Pal-SLNs showed even more pronounced effects on reducing the cytokine secretion compared to DSP (Figure 5e,f). The TNF-α levels were essentially identical at the level of 42 pg/mL for both DXMS-Pal-SLNs-1 and DXMS-Pal-SLNs-2, as compared to 102.45 pg/mL by the DSP treatment. In contrast to the reduction in the IL-6 level to119.02 pg/mL with DSP treatment, the IL-6 levels were found to be highly decreased to 14 pg/mL (DXMS-Pal-SLNs-1) and 12 pg/mL (DXMS-Pal-SLNs-2) with the treatment of the two SLN formulations via nebulization. These findings suggest that the nebulization of DXMS-Pal-SLNs leads to an effective accumulation within pulmonary macrophages for their therapeutic action as a glucocorticoid receptor agonist to reduce the pro-inflammatory cytokine secretion in the respiratory system.

**Figure 6:**
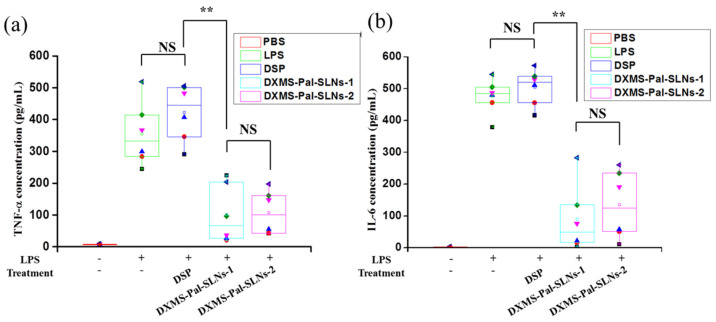
Determination of the levels of (**a**) TNF-α and (**b**) IL-6 in the BALF of mice experiencing the LPS-induced lung inflammation (with an LPS dose of 2.0 mg/kg) at 22 h after the treatments with DXMS-Pal-SLNs and DSP (DXMS 15 mg/kg in 6.0 mL PBS), respectively, via the nebulization administration (*n* = 6). NS *p* > 0.05 and ** *p* < 0.01.

During acute infections, pathogens such as viruses and bacteria can rapidly replicate or proliferate, therefore eliciting a robust immune response from the host. Such immune responses may cause significant damage, in particular to the infected areas, and may ultimately lead to mortality. To evaluate the efficacy of DXMS-Pal-SLNs via the nebulization route under severe inflammatory conditions, pneumonia-related inflammation in mice was induced by LPS of the double dose (2.0 mg/kg) and treated with DXMS-Pal-SLNs via nebulization at a DXMS dose of 15 mg/kg. As shown in Figure 6a,b, both the levels of TNF-α and IL-6 became more erratic and fluctuating for all the groups with the LPS dose of 2 mg/kg being employed, probably being related to the development of the tolerance to LPS [49]. Nevertheless, DXMS-Pal-SLNs at the DXMS dose of 15.0 mg/kg exhibited the sound capability of lowering the TNF-α and IL-6 secretion induced by the overdose stimulation of LPS in contrast to the negligible anti-inflammatory effect by the DSP treatment via nebulization. The results suggest that nebulized DXMS-Pal-SLNs could be an effective therapeutic approach for acute infection treatment as they mitigate the harmful effects of the immune responses, mostly probably because of the enhanced cellular uptake of the SLNs by alveolar macrophages via phagocytosis.

### 3.5. Biosafety of DXMS-Pal-SLNs

**Figure 7:**
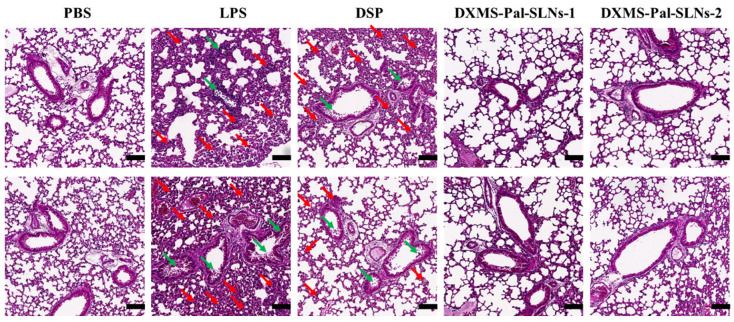
H and E-stained lung tissues of mice experiencing LPS-induced lung inflammation after the treatment with DSP, DXMS-Pal-SLNs-1 and DXMS-Pal-SLNs-2 via the nebulizer administration. The recruitment of alveolar macrophages and immunocytes in the alveolar sacs and bronchioles is highlighted by red and green arrows, respectively. The scale bars represent 50 μm.

Histopathological examination of lung tissues and other major organs with H and E staining was conducted to assess the biosafety of DXMS-Pal-SLNs. The pulmonary tissues with mice experiencing LPS-induced pulmonary inflammation showed extensive alveolar macrophage and other immunocyte infiltration in lung sections by H and E staining (Figure 7). The DSP group also showed similar severe conditions of pulmonary infiltration and recruitment of immunocytes. In contrast, the DXMS-Pal-SLNs exhibited well-preserved alveolar and bronchiole structures, similar to those in the PBS group in the absence of LPS-induced lung inflammation. These findings further substantiate the exceptional therapeutic efficacy of DXMS-Pal-SLNs when administered via nebulization for the treatment of pneumonia-related inflammation. As expected, there are no obvious damages in other major organs after the treatments with all groups, most probably because of the prevention of systemic side effects by the local anti-inflammation treatment to lung via nebulization (Figure S3). Moreover, DXMS-Pal-SLNs demonstrated high hemocompatibility by incubation with red blood cells, as shown in Figure S4. The H and E examination and hemolysis test strongly support the excellent bio-logical safety profile of the treatment throughout the study.

## 4. Conclusions

In this study, the SLN formulation of DXMS-Pal (DXMS-Pal-SLNs) was developed and evaluated in reducing the secretion of pro-inflammatory cytokines, namely TNF-α and IL-6, mainly by alveolar macrophages for pneumonia-associated inflammation treatment. The high loading efficiency of DXMS-Pal within SLNs (ca 94%), low drug leakage and excellent colloidal stability in PBS was attained. The recovery rates of DXMS-Pal-SLNs after nebulization were as high as approximately 90%, along with negligible changes in their mean particle sizes and PDIs. DXMS-Pal-SLNs showed a promising anti-inflammation effect even at a low dose of 5.0 mg/kg via intratracheal injection. As expected, the bioavailability was reduced in the DXMS-Pal-SLNs treatment via the administration route of nebulization compared to the intratracheal injection. This was caused by the inherent limit in the efficiency of mist inhalation, in particular when mice were tested in a closed chamber. Nevertheless, the DXMS-Pal-SLNs treatment via nebulization showed much enhanced efficacy against pulmonary inflammation compared to the DSP treatment via the same administration route. This is because of the high uptake efficiency of the SLNs by alveolar macrophages via phagocytosis in contrast to the entry of the DSP species into the cells via passive diffusion. The in vivo studies confirmed that DXMS-Pal-SLNs via nebulization administration effectively ameliorated LPS-induced pulmonary inflammation by reducing the secretions of TNF-α and IL-6 in the absence of pulmonary infiltration or immune cell recruitment to the inflammatory areas. DXMS-Pal-SLNs administered via the nebulization route demonstrate remarkable potential for treating acute lung infections. Given the absence of adverse side effects or hemolytic behavior, DXMS-Pal-SLNs may also benefit from intravenous administration, though further validation is required. Therefore, nebulized DXMS-Pal-SLNs demonstrated significant anti-inflammatory effectiveness, representing a promising therapeutic strategy for the local treatment of pulmonary inflammation.

## Figures and Tables

**Table 1:** DLS data, zeta potential and drug loading efficiencies (LE) and loading contents (LC) of DXMS-Pal-SLNs a,b.

Sample	DXMS-Pal	Size	PDI	ZP	LE/LC (DXMS-Pal)	LC (DXMS)
	(mg)	(D_h_ nm)		(mV)	(%)	(%)
DXMS-Pal-SLNs-1	4	183.4 ± 2.3	0.2 ± 0.03	−45.7	94.0 ± 1.1/15.8 ± 1.2	9.8 ± 0.7
DXMS-Pal-SLNs-2	6	195.4 ± 3.3	0.2 ± 0.02	−47.9	93.9 ± 3.2/23.3 ± 1.4	14.5 ± 0.9
DXMS-Pal-SLNs-3	8	196.1 ± 5.1 (precipitate over 7 days at 4 °C)	0.2 ± 0.02	−23.2	97.4 ± 2.6/26.3 ± 2.2	16.4 ± 1.3

a. DLS measurement was done in phosphate buffer (pH 7.4, I = 10 mM) at 25 °C.; b. Standard deviation is included with *n* ≥ 10.

**Table 2:** Recovery characterization of DXMS-Pal-SLNs after nebulization a,b.

Sample	Before Nebulization	After Nebulization			
	Volume (mL)	Volume (mL)	Recovery (%)	Size (D_h_ nm)	PDI
DXMS-Pal-SLNs-1	2.0	1.8 ± 0.05	90.2 ± 2.3	171.5 ± 1.9	0.2 ± 0.02
DXMS-Pal-SLNs-2	2.0	1.9 ± 0.07	86.2 ± 1.4	180.6 ± 1.7	0.2 ± 0.02

a. DLS study was examined in phosphate buffer (pH 7.4, I = 10 mM) at 25 °C.; b. Standard deviation is included with *n* ≥ 10.

## Data Availability

Data will be available upon request to corresponding authors.

## Supplementary Materials

The following supporting information can be downloaded at: https://www.mdpi.com/article/10.3390/pharmaceutics16070878/s1, Figure S1: Trachea photos of the LPS-induced pneumonia mouse before and after the treatment by intratracheal injection. Figure S2: Drug leakage profiles of DXMS-SLNs and DXMS-Pal-SLNs by the dialysis technique. Figure S3: H&E staining of major organs of the pneumonia mice after the various DXMS treatments. Figure S4: Hemolysis test of DXMS-PAL-SLNs. Table S1. Drug retention and leakage of DXMS-Pal-SLNs in PBS at 37 °C for 24 h.
