# Supplementary material for: Solid Lipid Nanoparticles Loaded with Dexamethasone Palmitate for Pulmonary Inflammation Treatment by Nebulization Approach
Source: Pharmaceutics. 2024 Jun 29;16(7):878. doi: 10.3390/pharmaceutics16070878 (PMC11279460; doi:10.3390/pharmaceutics16070878)
Supplement: Supplementary file 1 [file pharmaceutics-16-00878-s001.zip › pharmaceutics-3061357-supplementary.pdf]

pharmaceutics

Supplementary Materials

Solid Lipid Nanoparticles Loaded with Dexamethasone Palmitate for Pulmonary Inflammation Treatment by Nebulization Approach

Hsin-Hung Chen [1], Chen-Hsiang Sang [2], Chang-Wei Chou [1], Yi-Ting Lin [2], Yi-Shou Chang [1,2] and Hsin-Cheng Chiu [1,*]

[1] Department of Biomedical Engineering and Environmental Sciences, National Tsing Hua University, Hsinchu 30013, Taiwan ; bc8472@hotmail.com (H.-H.C.); tonychou@microbasetech.com (C.-W.C.); yishou@gmail.com (Y.S.C.)
[2] Department of R&D, Medical Division, MicroBase Technology Corp, Taoyuan City 33464, Taiwan; samnelsang@microbasetech.com (C.H.S.); edwinlin@microbasetech.com (Y.-T.L.)
* Correspondence: hscchiu@mx.nthu.edu.tw

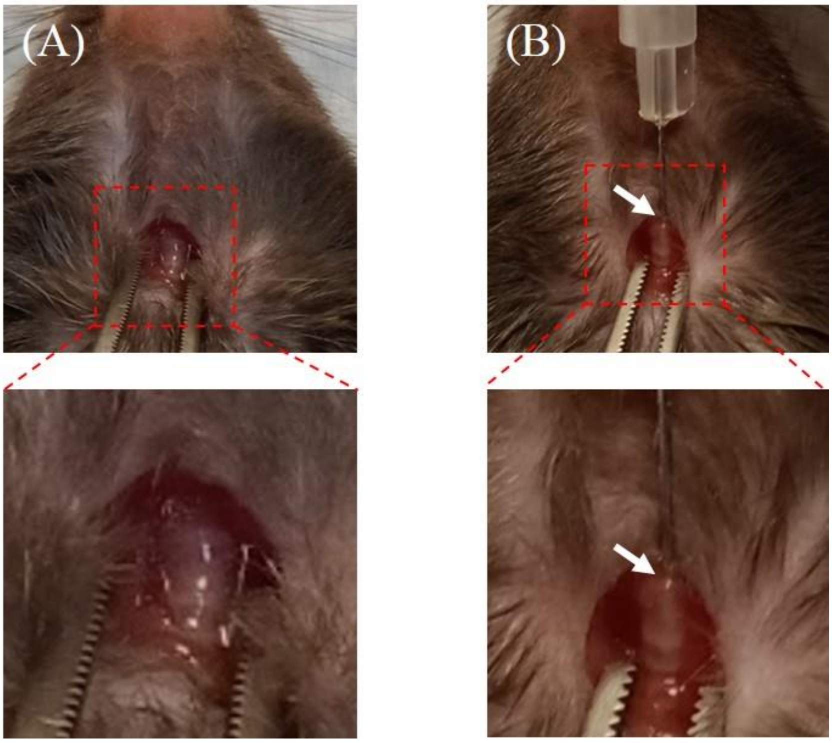

Figure S1. The trachea of the C57BL/6Jnarl male mouse experiencing LPS-induced pulmonary inflammation (A) before and (B) after the treatment with DXMS-Pal-SLNs in PBS by intratracheal injection (represented by white arrow).

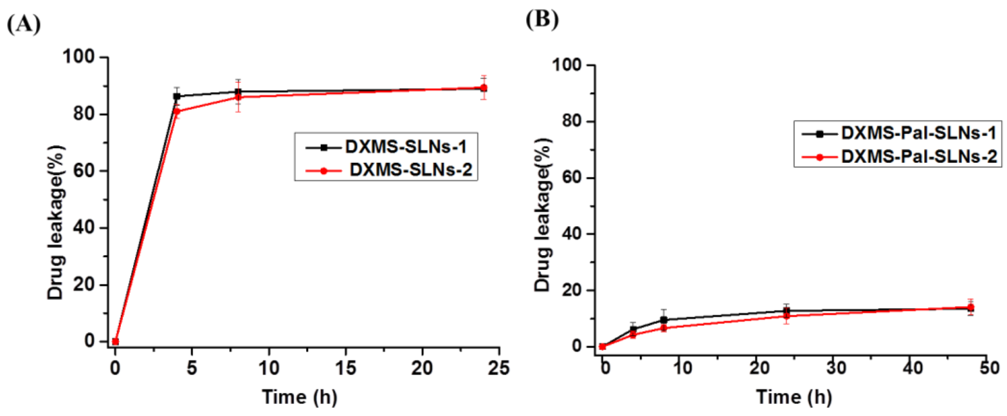

Figure S2. Drug leakage profiles of (A) DXMS-SLNs and (B) DXMS-Pal-SLNs in PBS at 37 °C. (n=3). The study was performed in PBS at 37 °C by dialysis technique. The preparation of DXMS-SLNs was essentially identical to that of DXMS-Pal-SLNs except that the amounts of DXMS in feed during the preparation were 2.5 mg in DXMS-SLNs-1 and 3.75 mg in DXMS-SLNs-2.

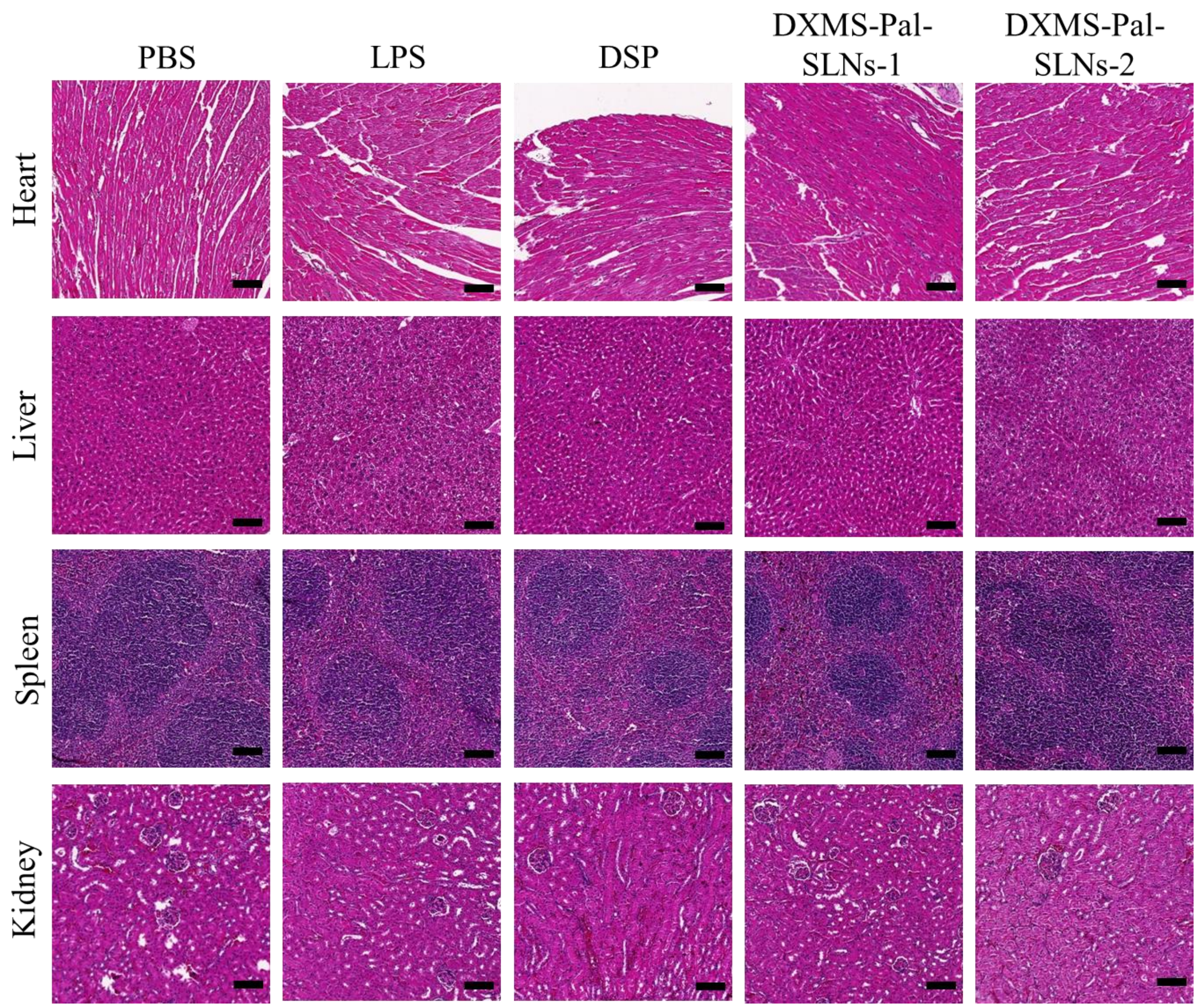

Figure S3. H&E staining of major organs of mice experiencing LPS-induced lung inflammation after treatments with DSP and DXMS-Pal-SLNs, respectively (lung not included). The scale bars are 50 μm.

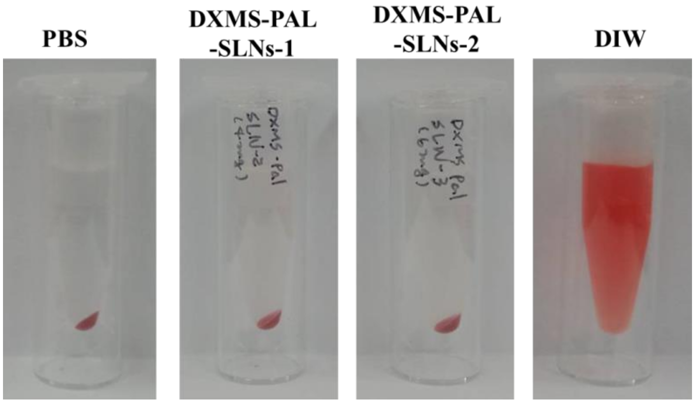

Figure S4. Hemolysis test of DXMS-PAL-SLNs. The red blood cells were co-incubated with PBS, DIW and DXMS-Pal-SLNs, respectively, for 6 h at 37 °C.

Table S1. Drug retention and leakage of DXMS-Pal-SLNs in PBS at 37 °C for 24 h.[a]

	DXMS-Pal-SLNs-1		DXMS-Pal-SLNs-2	
	µg	%	µg	%
Initial DXMS amount	297.5	100.0	385.8	100.0
Retention	256.7	86.3	360.8	93.5
Leakage	24.8	8.3	7.8	2.0

[a]The separation of DXMS-Pal and DXMS-Pal-SLNs was performed by gel permeation chromatography (Sephadex G-25 PD-10 column). The quantities of DXMS-Pal both released and retained were determined by UV/Vis spectroscopy at 238 nm.
